# Sequential bilateral cochlear implant: results in children and adolescents^☆^

**DOI:** 10.1016/j.bjorl.2018.07.008

**Published:** 2018-08-18

**Authors:** Gabriela Felix Lazarini Almeida, Marcella Ferrari Martins, Lucas Bevilacqua Alves da Costa, Orozimbo Alves da Costa, Ana Claudia Martinho de Carvalho

**Affiliations:** a Universidade de São Paulo (USP), Faculdade de Medicina (FM), São Paulo, SP, Brazil; b Alfa Instituto de Comunicação e Audição, São Paulo, SP, Brazil; c Universidade de São Paulo (USP), Faculdade de Odontologia de Bauru (FOB), Bauru, SP, Brazil

**Keywords:** Cochlear implant, Hearing, Hearing loss, Child, Speech perception, Implante coclear, Audição, Perda auditiva, Criança, Percepção da fala

## Abstract

**Introduction:**

The use of the bilateral cochlear implants can promote the symmetrical development of the central auditory pathways, thus benefiting the development of auditory abilities and improving sound localization and the ability of auditory speech perception in situations of competitive noise.

**Objective:**

To evaluate the ability of speech perception in children and adolescents using sequential bilateral cochlear implants, considering the association of these variables: age at surgery, time of device use and interval between surgeries.

**Methods:**

A total of 14 individuals between 10 and 16 years of age, who demonstrated surgical indication for the use of sequential bilateral cochlear implants as intervention in the auditory habilitation process, were assessed. The speech perception ability was assessed through sentence lists constructed in the Portuguese language, presented in two situations: in silence, with fixed intensity of 60 dB SPL, and in competitive noise, with a signal-to-noise ratio of +15 dB. The evaluation was performed under the following conditions: unilateral with the first activated cochlear implant, unilateral with the second activated cochlear implant and bilateral with both devices activated.

**Results:**

The results of the speech perception tests showed better performance in both silence and in noise for the bilateral cochlear implant condition when compared to the 1st cochlear implant and the 2nd cochlear implant alone. A worse result of speech perception was found using the 2nd cochlear implant alone. No statistically significant correlation was found between age at the surgical procedure, interval between surgeries and the time of use of the 2nd cochlear implant, and the auditory speech perception performance for all assessed conditions. The use of a hearing aid prior to the 2nd cochlear implant resulted in benefits for auditory speech perception with the 2nd cochlear implant, both in silence and in noise.

**Conclusion:**

The bilateral cochlear implant provided better speech perception in silence and in noise situations when compared to the unilateral cochlear implant, regardless of the interval between surgeries, age at the surgical procedure and the time of use of the 2nd cochlear implant. Speech perception with the 1st cochlear implant was significantly better than with the 2nd cochlear implant, both in silence and in noise. The use of the hearing aid prior to the 2nd cochlear implant influenced speech perception performance with the 2nd cochlear implant, both in silence and in noise.

## Resumo

**Introdução:**

A utilização do implante coclear bilateral pode promover o desenvolvimento das vias auditivas centrais de maneira simétrica, beneficiando, assim, o desenvolvimento das habilidades auditivas e melhorando a localização sonora e a habilidade de percepção auditiva da fala em situações de ruído competitivo.

**Objetivo:**

Avaliar a habilidade de reconhecimento de fala em crianças e adolescentes usuários do implante coclear bilateral sequencial, considerando a relação das variáveis: idade cirúrgica, tempo de uso do dispositivo e intervalo entre as cirurgias.

**Método:**

Foram avaliados 14 indivíduos entre 10 e 16 anos, que receberam indicação cirúrgica para a utilização do implante coclear bilateral sequencial como forma de intervenção no processo de habilitação auditiva. A habilidade de reconhecimento de fala foi avaliada por meio de listas de sentenças construídas na língua portuguesa, apresentadas em duas situações: no silêncio, com intensidade fixa de 60 dB NPS, e no ruído competitivo, com a relação sinal/ruído de + 15 dB. A avaliação foi feita nas seguintes condições: unilateral com o 1° implante coclear ativado, unilateral com o 2° implante coclear ativado e bilateral com ambos os dispositivos ativados.

**Resultado:**

Os resultados dos testes de reconhecimento de fala demonstraram melhor desempenho tanto no silêncio quanto no ruído para a condição implante coclear bilateral, quando comparado ao 1° implante coclear e 2° implante coclear isoladamente. Um pior resultado de reconhecimento de fala foi encontrado com o uso do 2° implante coclear isoladamente. Não foi encontrada correlação estatisticamente significante entre idade cirúrgica, intervalo entre as cirurgias e tempo de uso do 2° implante coclear e o desempenho de percepção auditiva da fala para todas as condições avaliadas. O uso do aparelho de amplificação sonora individual anterior ao 2° implante coclear beneficiou os resultados de percepção auditiva da fala com o 2° implante coclear, tanto no silêncio quanto no ruído.

**Conclusão:**

O implante coclear bilateral proporcionou melhor reconhecimento de fala em situações de silêncio e de ruído, quando comparado com o implante coclear unilateral, independentemente do intervalo entre as cirurgias, da idade cirúrgica e do tempo de uso do 2° implante coclear. O reconhecimento de fala com o 1° implante coclear foi significativamente melhor do que com o 2° implante coclear, tanto no silêncio quanto no ruído. O uso do aparelho de amplificação sonora individual anterior ao 2° implante coclear influenciou o desempenho de reconhecimento de fala com o 2° implante coclear, tanto no silêncio quanto no ruído.

## Introduction

Even considering all the technology applied to the current generation of cochlear implants and the fact that unilateral cochlear implant (CI) users show speech comprehension in silent environments and report improvements in the quality of life after the implantation, some difficulties in everyday situations have been reported by users of unilateral CI, such as sound localization and speech perception in noisy environments,^1^, ^2^ functions that require the binaural ability and may not be favored with the use of the unilateral CI.^3^

In recent years, the bilateral CI has been favored in the international context, and also as an alternative to promote binaural function. Studies have demonstrated speech perception improvement with bilateral CI, thus contributing to sound localization improvement^4^ and better speech perception in noise.^5^

The clinical and scientific literature has highlighted three reasons to implant the second ear: to ensure that the best ear is stimulated; to provide backup if device failure occurs, as well as to provide the benefits of binaural hearing, allowing an improvement in the localization of sound sources and in the auditory perception of speech in noisy environments.^6^

Bilateral CI surgery can be performed simultaneously or sequentially. In a simultaneous procedure, the two devices are implanted during a single surgical event; in a sequential procedure, the second device is implanted months or years after the first surgery. For sequential implantation cases, a question related to the impact of the time interval between the first and second surgeries regarding the post-surgical results has been discussed by researchers in the area.^7^

The bilateral CI is a reality in Brazil, and the patients’ interest in the second CI surgery among those who use unilateral CI is increasing. Therefore, these individuals undergo bilateral CI surgery sequentially, with intervals between surgeries that may be months or years, and it is of the utmost importance to evaluate the performance of auditory abilities in these individuals over time as well as to verify the aspects that can influence these results. However, studies in the scientific literature about the Brazilian experience of bilateral CI users are scarce.

In this context, it is of great importance to evaluate the benefits of bilateral CI, as well as the influence of the variables: age at the surgical intervention, time of device use, time interval between the surgeries and use of a hearing aid prior to the 2nd CI for speech perception to help in the indication of new candidates, as well as in the management of the speech-language therapy process.

## Objective

To evaluate the speech perception ability in children and adolescents with sequential bilateral CI and to analyze the influence of the variables: age at surgical procedure, time of device use, time interval between surgeries and hearing aid use prior to the 2nd CI for speech perception performance with the 2nd CI and with bilateral CI, in silence and in noise.

## Methods

A cross-sectional study was carried out in a cochlear implant center of the private health care network of the city of São Paulo, in 14 children and adolescents with sequential bilateral CIs, whose age ranged from 10 to 16 years and had received the 1st CI at a mean age of 29 months, with a mean interval between surgeries of 91 months. Nine individuals (64.3%) used hearing aid prior to the 2nd CI and five did not use it (35.7%).

Patient selection and evaluation procedures were initiated after the appropriate ethical processes: authorization was obtained from the institution where the study was carried out, approval by the Research Ethics Committee of the institution (n. 1754869) and signing of the Free and Informed Consent form by the patients’ parents or caregivers.

The following inclusion criteria were used to determine the study sample: severe to profound bilateral sensorineural hearing loss; first CI procedure up to 36 months of age; interval between the first and second CI surgeries ≥12 months; time of use of bilateral CI ≥ 12 months; effective use of bilateral CI (minimum of 8 h daily) and post-surgical medical and speech-language and audiology follow-up.

Speech perception assessment was performed by applying lists of sentences constructed for the Portuguese language,^8^ available in material recorded on a CD player and presented in a acoustic booth, using a two-channel audiometer connected to an amplifier in free field with the individual within 1 meter of the speaker. In the silence situation, the sentences were presented in a loudspeaker at 0° azimuth, at the fixed intensity of 60 dB SPL. For the noise situation, the sentences were presented in a loudspeaker at 0° azimuth, at the fixed intensity of 60 dB SPL, and the competitive noise (party noise) presented at 180° azimuth, with a signal-to-noise ratio (SNR) of +15 dB.^9^ The test results were obtained in percentage (%), with scores ranging from 0 to 100%, and the higher the score, the better the performance in the speech perception ability.

The speech perception results in silence were compared to those obtained in noise, at the conditions 1st CI, 2nd CI, and bilateral CI. Descriptive measures (mean and standard deviation) were used, as well as Pearson's correlation coefficient and the nonparametric Wilcoxon test for paired samples.

A descriptive analysis of the speech perception results with the 2nd CI and the bilateral CI in silence and in noise was performed between the individuals who used hearing aid prior to the 2nd CI and those who did not use conventional amplification. For the comparison of the results obtained in each group, a non-parametric analysis was performed, using the Mann–Whitney test for independent samples. Tukey's method was used to compare the means of the percentages of correct answers in silence and in noise between the groups.

## Results

**Figure 1:**
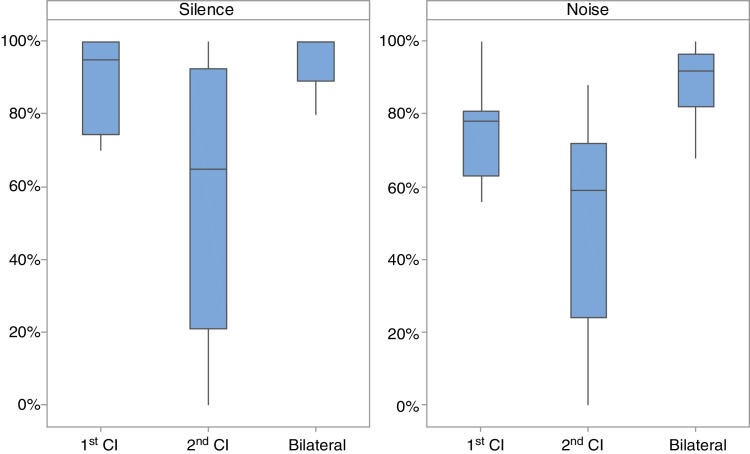
Percentage of correct answers in speech perception tests in silence and in noise in the three assessed conditions: 1st CI, 2nd CI and bilateral CI.

Better speech perception was observed with the use of bilateral CI, when compared to the results of the 1st CI and the 2nd CI separately, both in silence (respectively, *p* = 0.011 and *p* = 0.003) and in the presence of competitive noise (*p* = 0.002 and *p* = 0.001, respectively). The performance with the 1st CI was significantly better than with the 2nd CI, both in silence and in noise (Fig. 1).

The analysis of the association between the variables: surgical interval, age at surgical procedure for the 2nd CI and time of use of the 2nd CI for the speech perception results with the 2nd CI and with the bilateral CI showed no statistically significant correlation between the auditory performance with the 2nd CI and the bilateral CI and such variables, both in silence and in noise (Table 1).

**Table 1:** Correlation of the percentages of correct answers for speech perception in silence and in noise with the 2nd CI and bilateral CI and surgical interval, age at the 2nd CI surgery and time of use of 2nd CI.

	Situation	Interval between the 1st and the 2nd CI	Age at the 2nd CI surgery	Time of use of the 2nd CI
Speech perception with the 2nd CI	Silence	*r* = −0.28	*r* = −0.35	*r* = −0.06
		*p* = 0.339	*p* = 0.214	*p* = 0.852
	Noise	*r* = −0.33	*r* = −0.43	*r* = −0.03
		*p* = 0.251	*p* = 0.128	*p* = 0.922
Speech perception with bilateral CI	Silence	*r* = 0.50	*r* = 0.56	*r* = −0.48
		*p* = 0.067	*p* = 0.035	*p* = 0.084
	Noise	*r* = 0.38	*r* = 0.48	*r* = −0.52
		*p* = 0.178	*p* = 0.080	*p* = 0.054

CI, Cochlear Implant; *r*, Pearson's correlation coefficient; *p*, *p*-value.

**Figure 2:**
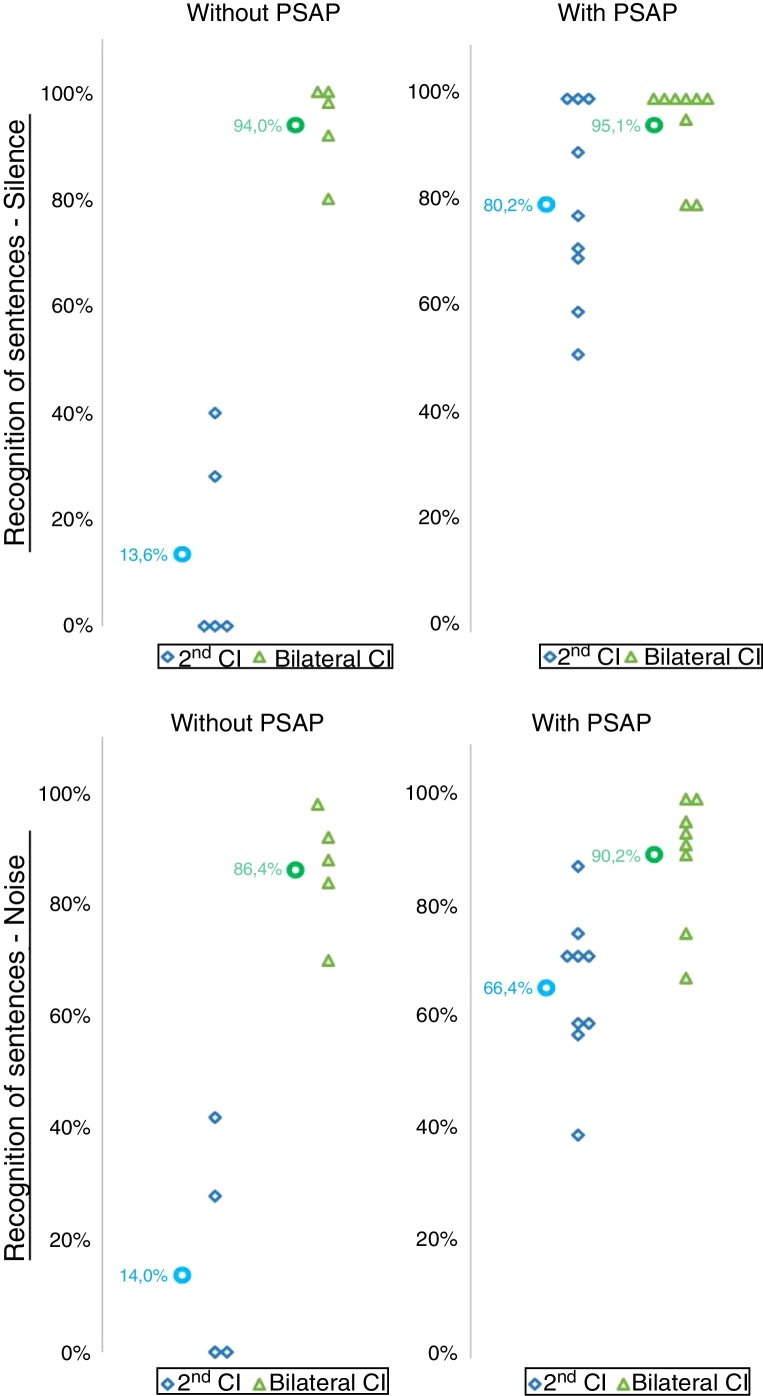
Individual and mean values of the percentage of correct answers in speech perception tests in silence and in noise with the 2nd CI and with the bilateral CI according to the use of hearing aid prior to the 2nd CI.

Regarding the speech perception ability in silence and in noise and the use of the hearing aid prior to the second CI surgery, a better performance with the second CI alone and with a bilateral CI, both in silence and in noise, was found for the group of individuals who used the hearing aid prior to the 2nd CI (Fig. 2).

A statistically significant difference was found for the speech perception results with the use of the 2nd CI, both in silence and in noise, for the group that used the hearing aid prior to the second surgery (*p* = 0.001 and *p* = 0.002, respectively). When evaluating performance with the bilateral CI, no statistically significant differences were found regarding speech perception between silence and noise in individuals who used and did not use the hearing aid prior to the second surgery (*p* = 0.606 and *p* = 0.364, respectively).

## Discussion

With the consolidation of the unilateral CI benefits, the possibility of indicating sequential bilateral CI has become part of the current clinical scenario, since a significant number of individuals who received CI in the first years of life may be potential candidates for sequential bilateral CI.

The scientific literature of the area has highlighted the benefits of bilateral CI in children and adults concerning the sound localization and speech perception abilities in competitive noise situations.

For the indication of sequential bilateral CI, a consensus has not yet been established regarding the desirable and necessary surgical interval to obtain the benefits of bilateral stimulation. Additionally, the age at the first CI surgery might be able to influence the speech perception results with bilateral CI.^7^, ^10^, ^11^

In the present study, all subjects received the 1st CI before the age of 3, during the period of maximal neuronal plasticity, thus allowing the development of the auditory skills that are essential for oral language development. Especially in the pediatric population, the surgical age at which the child was submitted to the 1st CI can be considered a predictor of auditory and language skill development.^12^, ^13^

Regarding auditory performance with the CI, all of the research subjects showed speech perception with the 1st CI (≥70%). However, despite the good performance with the unilateral CI, greater difficulty was found for the speech perception task in the presence of competitive noise, thus demonstrating the auditory challenges faced in situations of complex day-to-day listening that may have motivated the decision for a bilateral CI, even with a long interval between the surgeries.^14^

The results of the present study demonstrated that auditory performance with the bilateral CI was superior to that with the 1st CI and 2nd CI alone, with a statistically significant difference for the bilateral CI condition when compared to the other assessed conditions (Fig. 1).

These results corroborate the data found in the literature, of which auditory speech perception results are significantly better with the use of bilateral when compared to unilateral CI.^6^, ^10^, ^15^, ^16^, ^17^

Even with significant bilateral advantages, a statistically significant difference was found when comparing the performance between the 1st and the 2nd CI in silence and in noise, therefore suggesting an asymmetry between the auditory pathways and a predominance of the first implanted side in relation to the second one. These findings are in agreement with the scientific literature, in which children who used a unilateral CI for many years before receiving the 2nd CI showed auditory pathway and cortical function asymmetries, which resulted in a difference in auditory performance with the 2nd CI.^18^

Another aspect to be considered and that remains controversial in the clinical and scientific community refers to the ideal age for the 2nd CI, as well as the recommended interval between surgeries to achieve good hearing outcomes. In the present study, the interval between surgeries, the age at the 2nd CI surgery and the time of the 2nd CI use did not influence speech perception results in silence and in noise with the 2nd CI and with the bilateral CI (Table 1). These data are in agreement with other studies that described speech perception benefits, even in individuals with long intervals between surgeries.^7^, ^11^, ^17^, ^19^ It is likely that the age at which individuals were submitted to the 1st CI positively influenced the results of this study, since the stimulation provided by the 1st CI during the period of maximal neuronal plasticity allowed the auditory access and the development of an auditory system more prepared for the stimulation with the 2nd CI, even after a long surgical interval.^7^, ^20^, ^21^

On the other hand, the long interval between surgeries and the age at which the 2nd CI is performed can directly impact the results of auditory perception of speech with the bilateral CI, in addition to the fact that a late 2nd CI might not favor adequate neural connections, generating inferior results for the second implanted ear and a decrease in motivation regarding the use of another device, which did not attain the performance of the 1st CI. 12, 13, 19 However, it remains unclear whether there is a critical age and/or period for the indication of the second CI in this group of children with prelingual hearing loss who have used unilateral CI for many years.^15^, ^20^, ^21^

The use of hearing aid prior to the 2nd CI positively influenced the speech perception results with the use of the 2nd CI, since better results were found both in silence and in noise for the group that used the hearing aid (Table 1). For the bilateral CI, even though there was no statistically significant difference, a trend toward better speech perception performance in silence and in noise was observed in the group that used conventional sound amplification (Fig. 2).

In clinical practice, a great variability is observed regarding the use of the hearing aid, and it is not possible to define the percentage of patients who use combined electrical and acoustic stimulation after the 1st CI. In an international multicenter study, the total of bimodal users accounted for only 32% of the total.^22^

Although the scientific literature has not described in detail the influence of bimodal hearing on speech perception after the 2nd CI, authors have pointed out that the use of hearing aid prior to the 2nd CI can be considered a predictor for better speech perception with the 2nd and with the bilateral CI. They also emphasize that unilateral CI users should be encouraged to continue the use of sound amplification contralateral to the 1st CI to maintain auditory pathway stimulation.^20^, ^23^

For all subjects in the present study, improved speech perception skills with bilateral CI strengthened and consolidated the use of both devices in a continuous and effective manner. However, the indication for a sequential bilateral CI in children and adolescents with a long interval between surgeries should be considered in a cautious and careful manner.

Detailed information should be provided to the applicants of the sequential bilateral CI and their families considering the variability of the results found in the scientific literature. Even considering the encouraging results, it is essential to assess the bilateral advantages over time, as well as analyze the several factors that can influence the binaural benefits to be obtained from the use of bilateral CI.

## Conclusions

Better speech perception performance was observed with the use of bilateral CI, both in silence and in noise, when compared to the 1st CI and with the 2nd CI alone. Speech perception with the 1st CI was significantly better than with the 2nd CI, both in silence and in noise.

No statistically significant correlation was found between age at the time of the 2nd CI surgery, the time of use of the 2nd CI and the interval between the surgeries for speech perception in silence and in noise with the 2nd CI and the bilateral CI.

The use of a hearing aid prior to the 2nd CI positively influenced the speech perception performance with the 2nd CI, both in silence and in noise.

## Conflicts of interest

The authors declare no conflicts of interest.
